# Supplementary material for: Ex vivo intestinal permeability assay (X-IPA) for tracking barrier function dynamics
Source: NPJ Biofilms Microbiomes. 2023 Jul 3;9:44. doi: 10.1038/s41522-023-00409-0 (PMC10318059; doi:10.1038/s41522-023-00409-0)
Supplement: Supplementary file 1 — Supplementary Information [file 41522_2023_409_MOESM1_ESM.pdf]

Supplementary information - figure legends

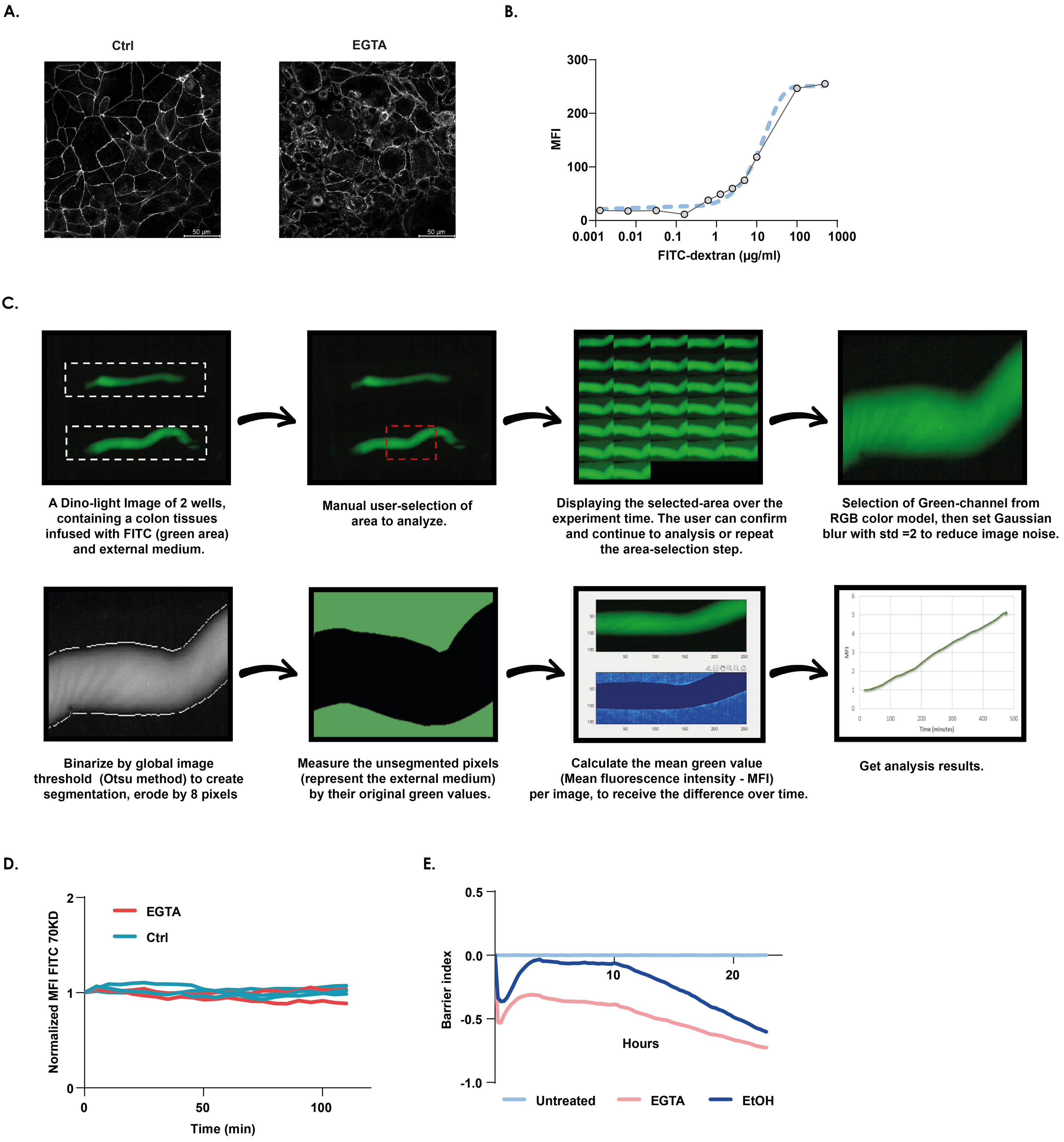
A.
Ctrl
EGTA
50 µm
50 µm
B.
MFI
300
200
100
0
0.001
0.01
0.1
1
10
100
1000
FITC-dextran (µg/ml)
C.
A Dino-light Image of 2 wells, containing a colon tissues infused with FITC (green area) and external medium.
Manual user-selection of area to analyze.
Displaying the selected-area over the experiment time. The user can confirm and continue to analysis or repeat the area-selection step.
Selection of Green-channel from RGB color model, then set Gaussian blur with std =2 to reduce image noise.
Binarize by global image threshold (Otsu method) to create segmentation, erode by 8 pixels
Measure the unsegmented pixels (represent the external medium) by their original green values.
Calculate the mean green value (Mean fluorescence intensity - MFI) per image, to receive the difference over time.
Get analysis results.
D.
Normalized MFI FITC 70KD
2
1
0
EGTA
Ctrl
0
50
100
Time (min)
E.
Barrier index
0.5
0.0
-0.5
-1.0
10
20
Hours
Untreated
EGTA
EtOH

Supplementary figure 1: Schematic representation of automated image analysis procedure using the X-IPA analyzer

(A) Confocal microscopy images showing disruptions to ZO-1 spatial organization (white) in Caco-2 cells incubated with EGTA (right) or sterile medium (left), for 1h. **(B)** Dynamic range of fluorescence detection by Dino-Lite digital microscope (dashed line - sigmoid nonlinear fit). **(C)** The tissue-images are loaded and cropped to mark the measurement-area. These coordinates include the intestinal tissue and the external surrounding medium. After user confirmation, the green channel of the RGB model is chosen and the images denoised using Gaussian filter, with a standard deviation of two. The denoised images are segmented by an automated threshold using Otsu's method from Gray-Level Histograms (Binarization), and the tissue segmentation is expanded by extra 8 pixels. Per image, the medium pixels (area outside of the segmentation) are measured, and their mean fluorescence value is calculated. These mean fluorescence values are shown in a plot, demonstrating the MFI (mean fluorescence intensity) changes over time. **(D)** Single colon time traces showing normalized MFI of the extraintestinal medium in tissues infused with EGTA or sterile medium (Ctrl), with 70kD-FITC-dextran. **(E)** Normalized TEER values of Caco-2 cells incubated with EtOH (blue) or EGTA (red), over time.

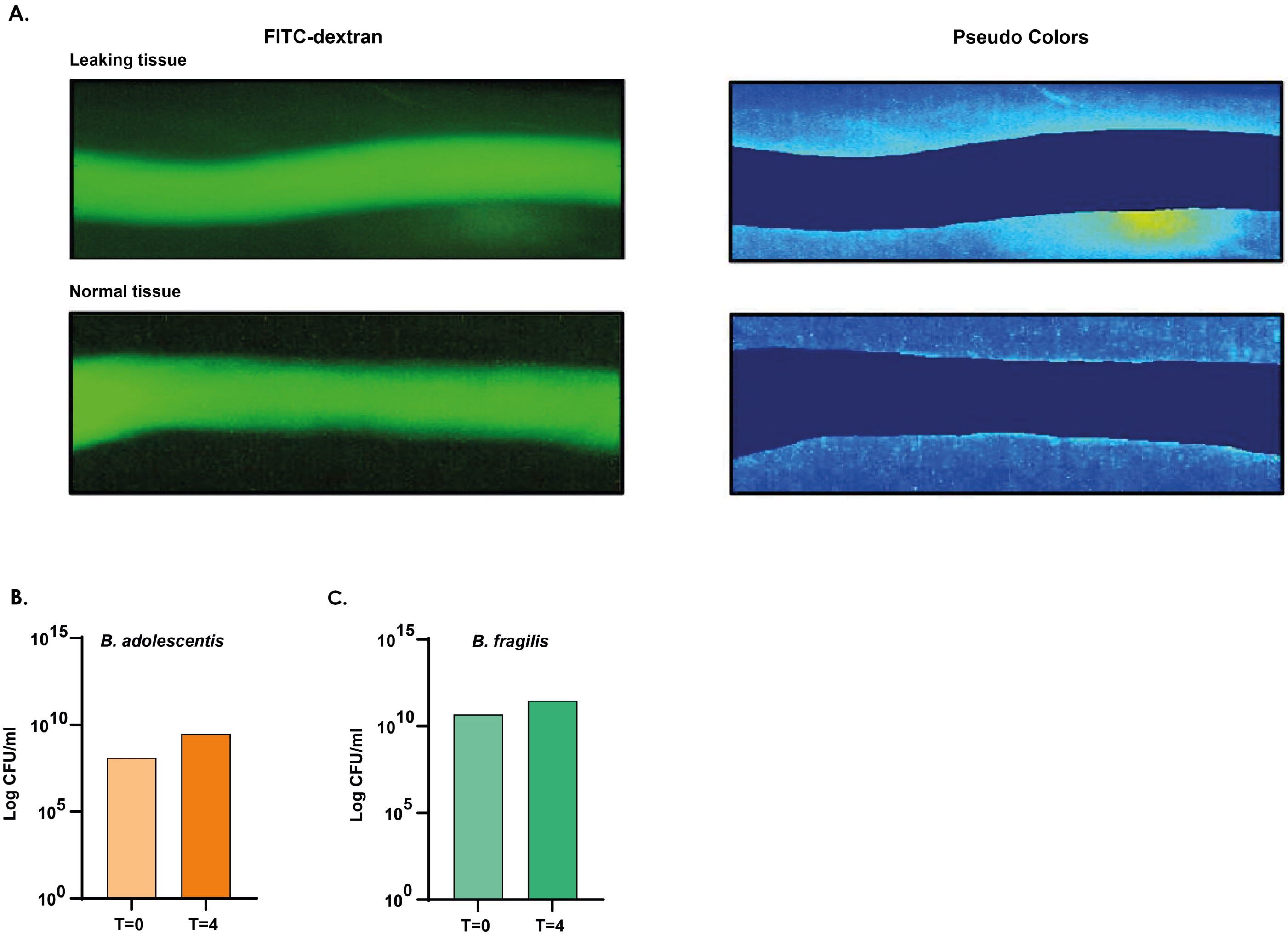
A.
FITC-dextran
Pseudo Colors
Leaking tissue
Normal tissue
B.
B. adolescentis
Log CFU/ml
10^{15}
10^{10}
10^{5}
10^{0}
T=0
T=4
C.
B. fragilis
Log CFU/ml
10^{15}
10^{10}
10^{5}
10^{0}
T=0
T=4

Supplementary figure 2: Quality control – detection of abnormal extraintestinal fluorescence

(A) The X-IPA analyzer software allows the user to identify abnormal MFI values, which may result from tissue damage during surgery and not from increased intestinal permeability. The interactive pseudo-colors allow the user to detect the leakage of FITC-dextran from the tissue throughout the time lapse movie. As an example, the upper images show a damaged, leaky tissue. The leaky area appears stronger according to the pseudo-colors scale and concentrated within a defined region of the extraintestinal medium. The bottom images show a normal, intact tissue, that is surrounded by medium with normal fluorescence distribution. **(B-C)** Microbial load (CFU/mL) of *B. adolescentis* **(B)** or *B. fragilis* **(C)**, measured before and after 4h infusion into gut organ cultures. Bacterial samples where cultured for 48h under anaerobic condition.

Supplementary video 1: EGTA rapidly increases gut permeability

A time lapse movie showing colon tissues infused with FITC-dextran for 8h, reveals a dramatic increase of fluorescence in the medium of the EGTA infused tissues compared to Ctrl (sterile medium only). Here, 3 EGTA-infused tissues and 3 medium-infused tissues were measured. The FITC-dextran animation shows the increasing of fluorescence in a representative tissue, as captured by the Dino-light microscope lens over time. The Pseudo-colors animations shows the increasing of fluorescence in the tissue, as analyzed by the X-IPA analyzer software over time. Frames are separated by 15min.

Supplementary video 2: EGTA increases small intestine permeability

A time lapse movie showing small intestinal tissues infused with FITC-dextran for 4h, reveals an increase of fluorescence in the medium of the EGTA infused tissue compared to Ctrl (sterile medium only). The FITC-dextran animation shows the increasing of fluorescence, as captured by the Dino-light microscope lens over time. The Pseudo-colors animations shows the increasing of fluorescence in the tissue, as analyzed by the X-IPA analyzer software over time. Frames are separated by 10min.

Supplementary video 3: EtOH increases gut permeability

A time lapse movie showing colon tissues infused with FITC-dextran for 4h, reveals a dramatic increase of fluorescence in the medium of the EtOH infused tissue compared to Ctrl (sterile medium only). The FITC-dextran animation shows the increasing of fluorescence in a representative tissue, as captured by the Dino-light microscope lens over time. The Pseudo-colors animations shows the increasing of fluorescence in the tissue, as analyzed by the X-IPA analyzer software over time. Frames are separated by 10min.

Supplementary video 4: Putrescine rapidly increases gut permeability, in a dose-dependent manner

A time lapse movie showing colon tissues infused with FITC-dextran for 4h, reveals a dose-dependent increase in extraintestinal medium fluorescence following infusion of putrescine compared to Ctrl

(sterile medium only). The FITC-dextran animation shows the increasing of fluorescence in a representative tissue, as captured by the Dino-light microscope lens over time. The Pseudo-colors animations shows the increasing of fluorescence in the tissue, as analyzed by the X-IPA analyzer software over time. Frames are separated by 5min.

Supplementary video 5: *B. adolescentis*, but not *B. fragilis*, rapidly increases gut permeability

A time lapse movie showing colon tissues infused with FITC-dextran for 4h, reveals an increase in extraintestinal medium fluorescence following infusion of *B. adolescentis* compared with tissues infused with *B. fragilis* or Ctrl (sterile medium only). The FITC-dextran animation shows the increasing of fluorescence in a representative tissue, as captured by the Dino-light microscope lens over time. The Pseudo-colors animations shows the increasing of fluorescence in the tissue, as analyzed by the X-IPA analyzer software over time. Frames are separated by 5min.
